# Response of Rambler Roses to Changing Climate Conditions in Urbanized Areas of the European Lowlands

**DOI:** 10.3390/plants10030457

**Published:** 2021-02-28

**Authors:** Marta Joanna Monder

**Affiliations:** Department of Dendrological Collections, Polish Academy of Sciences Botanical Garden—Center for Biological Diversity Conservation in Powsin, Prawdziwka 2, 02-973 Warsaw, Poland; m.monder@obpan.pl

**Keywords:** climate change, frost damage, growing season, historical roses, phenology, precipitation, temperature, urban greening, winter hardiness

## Abstract

Climate change affects the possibility of crop production and yield and disrupting the maintenance of crop biodiversity, including ornamentals. Warsaw is located in a temperate zone with mixed continental and oceanic climate influences. This research examines the response of once-blooming rambler roses to changing climate conditions in connection with their frost resistance and ornamental value. The 15 selected rambler rose cultivars were observed in the years 2000–2016 in the Polish Academy of Sciences Botanical Garden—Center for Biological Diversity Conservation in Powsin. Damage to shrubs caused by frost, the timing of bud break, leaf development, and initial, full, and final flowering were recorded. We show that changes in phenology and frost damage were the effect of weather conditions in the autumn–winter–spring period. Frost damage influenced the flowering and growth of plants in different ways, depending on the extent of required pruning. The cultivars most highly tolerant to frost damage were: “Lykkefund”, “Polstjårnan”, and “Semiplena”. During the final years (2014–2016), due to mild winters, all of the studied rose cultivars could be used for a wider range of applications than previously (2000–2006 and 2009–2013). Their reintroduction helped to maintain biodiversity of old cultivars, which makes these roses a proposal for the lowlands of Central Europe.

## 1. Introduction

Knowledge of the phenology of wild and cultivated plants is important for horticultural crop production, meteorological sciences, and botany [1,2]. Plant phenology is a source of knowledge of periodic biological events affected by the environment and also the most reliable bioindicator [3,4]. It reflects biological and physical systems independently [5]. Changes in springtime phenological events of perennial [6,7,8] and woody plants have often been documented [3,5,7,9] and are more consistent in direction and magnitude than changes in summer and autumn phenophases [4,5,7,9,10]. Zheng et al. [5] selected 11 phenophases in nine woody species, namely, bud expansion, bud burst, first flowering, 50% of full flowering, end of flowering, first leaf, 50% of full leaf expansion, beginning of leaf coloring, end of leaf coloring, beginning of leaf fall, and end of leaf fall [5]. The beginning dates of spring and summer phenophases advanced with time, while the start of autumn and winter phenophases became delayed. These changes were significantly correlated with temperature [5]. Changes in climate can affect bud dormancy and cold hardiness, which are critical adaptations for the survival of winter cold stress by perennial plants of the temperate zone, *Vitis* species among them [11]. Bud dormancy allows perennial and woody plants to survive the winter in temperate climates [12].

To examine the effects of climate change, botanical gardens make standard phenological observations of many plant taxa growing on their area limiting the number of factors that might alter long term changes [13]. Their behavior can provide insight into how species will respond in the wild in terms of, e.g., changes in flowering, leaf-out times, and fruiting [13]. The same principles can be applied to crops and ornamental plants. Botanical gardens located in large urban areas have tended to warm more rapidly than surrounding areas because of the urban heat island effect [13]. Moreover, the effects of gas pollution on many plant traits, such as their phenology, seem minor in relation to the effects of temperature, light, and precipitation [14]. Botanical gardens have a unique set of resources, including controlled growing conditions, which allows them to host important climate change research that cannot be easily undertaken elsewhere. Due to hosting large collections of plants from a wide range of areas, they are in the position to address many questions related to climate change that are often too difficult to examine at any other location [15]. Observations in botanical gardens are recorded annually or periodically by experienced staff, and these records can also be used to track changes in anatomy or physiology [13].

The rapid climatic changes observed over several dozens of years indicate that the average temperature has risen and the vegetation season has been slightly prolonged in the European area [16]. These changes influence the diversity and distribution of species, and consequently ecosystems and biodiversity. It is estimated that ca. 32% of European plant species present in 1990 will disappear by 2050. These species take up 44% of the modeled European area. The distribution of natural species is projected to shift towards the north-east [16]. Due to climatic change, some species will no longer be able to grow at their present locations due to lack of temperature tolerance, water stress, competition with other plant species, or changes in patterns of herbivory [16,17,18].

The import of new species to nurseries and, in consequence, to the market poses a risk of their emergence as invasive species in the local environment and landscape, particularly in rural areas [19]. The most decisive climate factors limiting the number of non-native plant species able to grow are not only frost resistance and autumn–winter–spring conditions but also the ability to grow in a changing climate [19,20]. During the years 2002–2016 1781 new species and cultivars were introduced in Polish nurseries associated with the Polish Nurserymen Association [21]. Most of them come from a higher zone—6B in the USDA codification [22]—and originate from, e.g., Western Europe or Asia [19]. However, agriculture, horticulture, and forestry are the main sources of alien plant expansion. Such species can become invasive and lead to the extinction and reduction of native species [23].

The use of cultivars in gardens limited the introduction of new potentially invasive species to the market by what is safer for maintaining the biodiversity of the environment; however, some are expansive, and their seed production should be evaluated in this regard. Their invasiveness needs to be considered on the basis of the evaluation of the entire life cycle of the cultivar and its offspring [24].

Roses are one of the oldest cultivated crops [25] and most important ornamental plants [26,27]; moreover, they have been significant in many fields of human life, both in Asia and in Europe, for thousands of years. The cultivated species, varieties, and cultivars of roses are arranged in several dozen groups in terms of origin and type of growth [25]. Roses can be ordered in terms of type of growth into the following groups: Hybrid Teas, Floribundas, Polyanthas, Miniatures, Rambler and Climbers, Shrubs, and Ground Cover [25,28]. The classification of basic groups of historical rambler roses in terms of their origin includes, e.g., Hybrid Wichurana, Hybrid Setigera, and Hybrid Multiflora [25,28]. The healing properties of roses have been appreciated and documented for centuries. The production of petal oil, the most important rose ingredient used in perfumery and the cosmetic industry, is estimated to continue expansion [3]. Rose oil also has applications in pharmacology, in which it is used for its anti-HIV, antibacterial, antioxidant, hypnotic, antidiabetic, and relaxant effects [27]. Climbing roses are a very diverse group of varied origin and different decorative values [21,25,28]. They are traditionally used in parks, courtyards, squares, estate greenery, and gardens. They do not require much space and are able to vine up walls in very narrow streets, right next to buildings and small courtyards [21,28] like other climbing plants [29]. Roses planted in restaurant and café gardens make sitting at nearby tables more pleasant. The importance of roses as part of the greenery in the city is not to be questioned [21,28]. Moreover, most rose cultivars produce few or no seeds and are not expansive [21,25,28].

Historical roses were known and cultivated before World War II [28,30,31,32,33]. They deserve special attention and should be used to revitalize historical properties and urban greeneries and maintain the biodiversity and heritage of garden plants more often [11,30,32,34].

Warsaw, Poland, is located in the Central European Mazovian Lowland [35]. Over 1000 taxa were gathered in the National Collection of Rose Cultivars in the Polish Academy of Sciences Botanical Garden—Center for Biological Diversity Conservation in Powsin. Among them, more than 200 taxa of different origin are historical roses. Maintaining collections of plants, including ornamental plants, is an important task for botanical gardens due to its contribution to the preservation of biodiversity. The gene pool of roses is in need of expansion—its currently narrow range may lead to so-called genetic erosion [36,37,38,39]. This research can help to encourage the maintenance of biodiversity of the genetic pool of old garden roses and to implement the provisions of the Convention on Biological Diversity (CBD) drawn up in Rio de Janeiro on 5 June 1992.

Recent changes in climate are worrisome [40,41], and their influence on cultivated ornamental plants, including historical cultivars, is little known. The aim of this study was to compare different rose cultivars with each other and to perform long-term monitoring of selected phenology events to gain a better understanding of the effect of regional climate change. The observations presented here were conducted in order to evaluate the response in phenology of different rose cultivars to temperature over a period of time (from 2000 to 2016) and the ornamental value and frost resistance of 15 rambler rose cultivars in changing climate conditions. Moreover, the interrelationships between the phenology stages were analyzed.

## 2. Material and Methods

### 2.1. Place of Research 

Warsaw, Poland, is located in the temperate zone, with mixed continental and oceanic climate influences. The Polish Academy of Sciences Botanical Garden—Center for Biological Diversity Conservation in Powsin (52.6° N, 20.5° E) is located in the Middle Vistula mesoregion, separated from the Warsaw Plain by a high fluvial terrace at the border of a post-glacial height. The surface is covered with eolic sand fields and, in some areas, with dunes and partially with dust deposits created by periglacial and eolic processes [35]. Warsaw is classified as belonging to the USDA zone 6B with a minimal average temperature between −20.6 and −17.8 °C [22].

### 2.2. Weather Conditions

**Figure 1:**
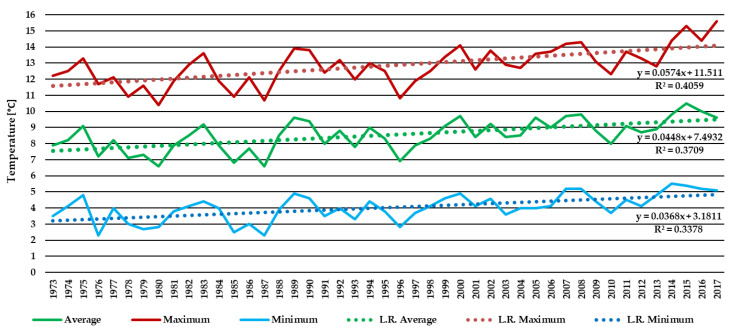
The mean monthly temperatures (°C) (average, minimal, and maximal) in the years 1970–2016 measured at the Warsaw-Okęcie meteorological station (10 km in a straight line from PAS Botanical Garden) and the linear trend lines in this period.

The average monthly temperatures in the years 1970–2016 measured in the Warsaw-Okęcie meteorological station (10 km in a straight line from PAS Botanical Garden) are shown in Figure 1 and expose the climate warming of the Warsaw region.

**Figure 2:**
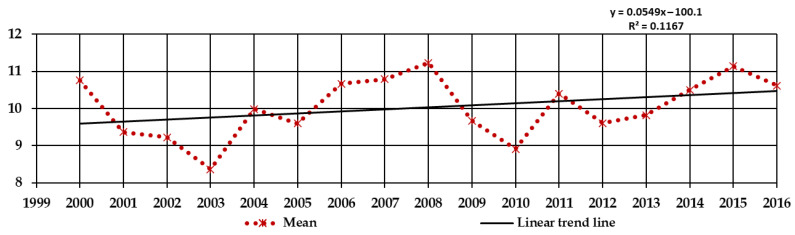
The total yearly mean air temperatures (°C) in the years 2000–2016, measured in the PAS Botanical Garden CBDC in Powsin, and the linear trend line of increase the temperature in this period.

The weather conditions were observed in the years 2000–2016 in the PAS Botanical Garden CBDC in Powsin. The end of the 20th century experienced many extreme climate events, such as significant warm and dry periods in spring and summer and catastrophic rainfalls [41,42]. Especially adverse weather conditions for roses occurred in autumn-winter-spring (2002/2003, 2005/2006, 2009/2010, 2010/2011, 2011/2012, 2012/2013, 2014/2015, and 2015/2016). The lowest minimal temperatures (below −15 °C) were noted in January: 2003 (−22.0 °C), 2009 (−22.5 °C), 2010 (−25.0 °C), 2013 (−15.1 °C), 2014 (−17.0 °C), and 2016 (−18.4 °C); and February: 2011 (−18.1 °C) and 2012 (−26.0 °C). Low temperatures were also, though rarely, noted in March, e.g., 2013 (−14.2 °C). Heavy snowfall occurred every year since the winter of 2009/2010. The course of changes in temperature took different forms, including, e.g., sudden temperature jumps in spring (2002/2003, 2012/2013, and 2015/2016), early frost (2002/2003, 2009/2010, 2010/2011, 2012/2013, and 2014/2015), or late frost (2005/2006, 2011/2012, and 2013/2014), a period of frost after a few days of warming (2002/2003, 2014/2015, and 2015/2016), long periods of frost (2002/2003, 2005/2006, 2011/2012, and 2012/2013), sudden spring warming (2009/2010, 2011/2012, 2012/2013, and 2013/2014), and rapid changes and high 24-hour amplitudes of temperature, especially in April (Figure S1). Regression analysis showed an increase in mean annual temperatures in the period of research, confirming the global warming trend (Figure 2). The monthly precipitation and average monthly temperatures in the years 2000–2016 based on measurements carried out in the PAS Botanical Garden CBDC are presented in Supplementary materials in Figures S2 and S3.

### 2.3. Plant Material

The observations were conducted in the National Collection of Rose Cultivars of the Polish Academy of Sciences Botanical Garden—Center for Biological Diversity Conservation in Powsin. The shrubs of 15 once-blooming cultivars of different origin and belonging to different groups [21,25,28] were selected for this study (Table 1).

One-year shrubs budded on rootstocks (*Rosa canina* L.) were planted in this period in a space that provided them with appropriate growth conditions, ample sunlight, supports, and soil enriched with organic materials with a pH of 6–6.5. Agrotechnical procedures were carried out according to the current technology [21,25,28]. The shrubs were fertilized with organic granular manure (50-60 g/pro plant) and the organic fertilizer “Azofoska” (4–6 kg/m^2^; “Grupa INCO”, Poland) every year in the spring after pruning. The roses were not irrigated from the second year after planting onward. The bases of shrubs were hilled up with bark for winter, and 2–3 protective sprayings of the plants against pests and fungal diseases were carried out every year. The shrubs were pruned every year, first in spring and then once more in the summer after overblooming [25].

### 2.4. The Evaluation of Plants

The roses were evaluated every year between 2000 and 2016.

Every spring, damage caused by frost was recorded according to the following scale:

0—undamaged plants;

1—darkened vascular bundles on shoots, but buds still develop;

2—frost-damaged leaf-buds;

3—frost-damaged one-year-old shoot tips;

4—frost-damaged one-year-old shoots or only their living bases;

5—frost-damaged also 2-year-old and older shoots;

6—the shoots frost-damaged to the ground level;

7—complete plant frost damage (no signs of regeneration). 

The plants’ developmental cycle is subdivided into clearly recognizable longer-lasting principal growth stages described using numbers from 0–9 in ascending order. Certain stages may be shifted or omitted in some species [2]. The BBCH-scale is a system for the uniform coding of phenologically similar growth stages of plant species. The BBCH is an abbreviation of **B**iologische **Bundesanstalt**, Bundesortenamt, and **CH**emische Industrie [2]. The work of Meier et al. [1] described the principal phenological stages of wild and cultivated roses, also in relation to the production system [1]. The same scale was used in this research.

During the winter, early spring, and summer periods, observations of vegetative bud break and flowering were made and recorded twice a week (every 3–4 days). The first stages (07 and 11 BBCH) were observed on the middle part of uncut one-year shoots if these were not frost-damaged. It should be noted that after low cutting the development of buds was delayed and noted on the remaining shoots. The flowers present on the middle part of typical one-year mature shoots and the timing of their appearance were noted once they represented a stage in which the large majority of the flowers were flowering according the BBCH scale.

**Figure 3:**
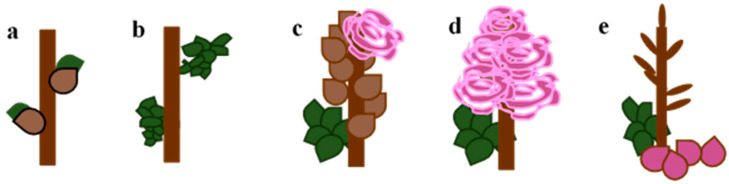
The chosen phenology stages of observation of shrubs: (**a**) BCH 07—beginning of bud breaking, first green leaf tips visible; (**b**) BBCH 11—first leaf pair unfolded, not yet at full size, leaves are light green and/or bronze; (**c**) BBCH 60 601—beginning of flowering: about 10% of flowers open; (**d**) BBCH 63 605—full flowering: at least 50% of flowers open; and (**e**) BBCH 69 609—end of flowering: all petals have fallen.

All the chosen phenology stages of observation of rambler roses were conducted using the BBCH-scale (Biologische Bundesortenamt, CHemische Industrie) described for rose cultivars [1] starting after their resting period of winter dormancy (00 BBCH):

07—beginning of bud breaking, first green leaf tips visible;

11—first leaf pair unfolded, not yet at full size, leaves are light green and/or bronze;

60 601—beginning of flowering: about 10% of flowers open;

63 605—full flowering: at least 50% of flowers open;

69 609—end of flowering: all petals fallen (Figure 3).

Although the bud breaking process involves several stages up until full leaf development [1,2], and despite the one-year shoots being a few meters long, only the initial points of breaking bud dormancy were recorded and considered in this work. For the above reasons, the following time intervals have been determined and are used in the paper in Figures 6 and 7 in results.

When the buds on unpruned shoots begin to break, the first green leaf tips are visible (BBCH 07) in rambler roses: VE—1–10 March; E—11–20 March; SE—21–31 March; M—1–10 April; SL—11–20 April; and L—21–30 April.

When the first leaf pair has unfolded, not yet at full size, leaves are light green and/or bronze on unpruned shoots (BBCH 11) of rambler roses: VE—21–31 March; E—1–10 April; SE—11–20 April; SL—21–30 April; and L—after 30 April.

The timings of flowering entered into each recorded phenological stage were converted to the number of days since the 25 May. The resulting timing represents an average recorded from each exemplar of the evaluated cultivar.

**Figure 4:**
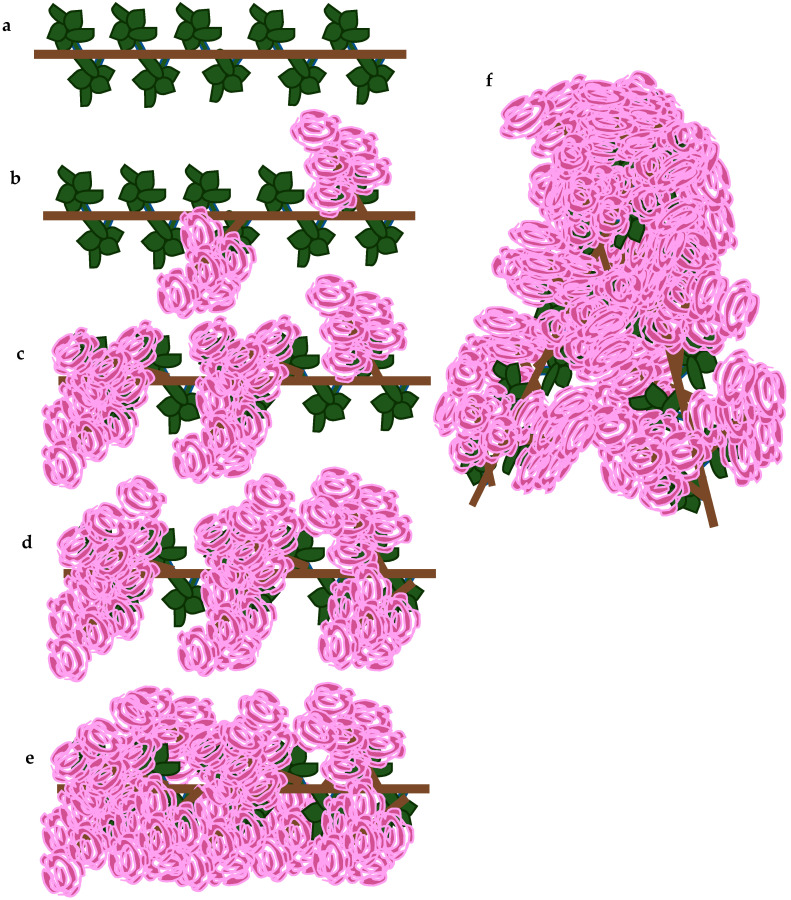
The abundance of flowering on a scale 0–5, where: (**a**) 0—lack of flowers; (**b**) 1—some inflorescences on shoots; (**c**) 2—no more than 5 inflorescences on 1 m of shoot, poorly flowering; (**d**) 3—inflorescences with numerous flowers, flowering with average abundance; (**e**) 4—more than 5 inflorescences on 1 m of shoot, the flowering plentiful and long; and (**f**) 5—exceptionally plentiful and long flowering, the flowers densely cover the plant.

The abundance of flowering was evaluated on a scale 0–5 (Figure 4), where:

0—lack of flowers;

1—some inflorescences on shoots;

2—no more than 5 inflorescences on 1 m of shoot, poorly flowering;

3—inflorescences with numerous flowers, flowering with average abundance;

4—more than 5 inflorescences on 1 m of shoot, the flowering plentiful and long;

5—exceptionally plentiful and long flowering with flowers densely covering the plant.

The height of the plants was noted in spring after pruning and at the end of the vegetation season. It should be noted that the height of plants was not equal to the length of their shoots. The height of supports is given in Table 1.

### 2.5. Statistical Analysis

The results of frost damages, bud break (BBCH 07), leaf development (BBCH 11), the date of the start of flowering, and its abundance were analyzed by using an analysis of variance (one-way ANOVA), and Duncan’s honest significant difference test was used to determine the significance of differences between the means (*p* ≤ 0.05). Additionally, Pearson correlations analyses between frost damages, bud break (BBCH 07), leaf development (BBCH 11), the date of the start of flowering, and its abundance were performed for the all cultivars. SPSS (IBM Statistics) software was used.

Moreover, the correlations were examined for each month from October to April, in selected seasons (2005/2006, 2009/2010, and 2015/2016) with long and frosty autumn–winter–spring periods, between average monthly temperature and:

1. Frost damage;
2. Periods when the buds on unpruned shoots are at the beginning of breaking and the first green leaf tips are visible (BBCH 07);
3. The bud on unpruned shoots are beginning to break, the first green leaf tips are visible, the first leaf pair has unfolded, not yet at full size, leaves are light green and/or bronze (BBCH 11);
4. The beginning of flowering: about 10% of flowers open (BBCH 60 601).

Additionally, the correlations between minimum air temperatures throughout the month and frost damage were examined. All years of observation were analyzed (2000–2016) [43].

STATISTICA 10 (StatSoft, Cracow, Poland) software was used.

## 3. Results

### 3.1. Frost Damages

**Figure 5:**
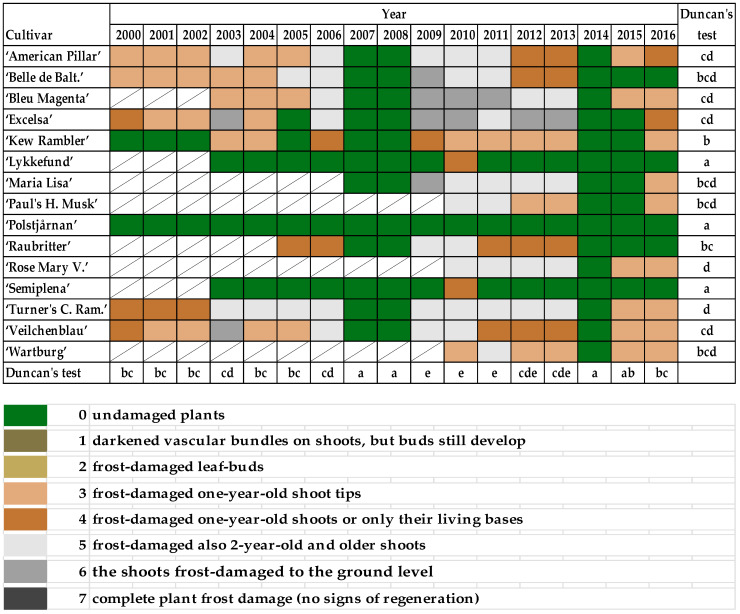
Frost damage of rambler roses according to the following scale: 0—undamaged plants; 1—darkened vascular bundles on shoots, but buds still develop; 2—frost-damaged leaf-buds; 3—frost-damaged one-year-old shoot tips; 4—frost-damaged one-year-old shoots or only their living bases; 5—frost-damaged also 2-year-old and older shoots; 6—shoots frost-damaged to the ground level; 7—complete plant frost damage (no signs of regeneration). Different letters indicate significant differences between cultivars and years. The Duncan’s test (α = 0.05) was used.

The frost damage of rambler roses is shown in Figure 5. Among the 15 varieties, only the shrubs of “Polstjårnan” did not have any damage every year. Moreover, “Lykkefund” and “Semiplena” had their one-year-old shoots or living bases frozen (5) only in the season 2009/2010 (the lowest minimal temperature was noted on 27.01. at −25 °C and 19.12. at −18.9 °C). “Kew Rambler” was also characterized by high resistance to frost—only one-year shoot tips were damaged. Lowered frost resistance was shown by “Rose Mary Viaud” and “Turner’s Crimson Rambler”. None of the cultivars experienced frost damage in the years 2007, 2008, and 2014 (Figure 5).

Correlation analysis of the Ramblers in terms of frost damage showed a strict relationship between the average monthly temperature and the scale of frost damage in all cultivars.

Lower average temperature in October, January, and February was correlated with high frost damage in most cultivars, with the exception of “Polstjårnan” and “Lykkefund”. Higher average temperature in November was correlated with high frost damage for the “Kew Rambler” and “Semiplena”, while in March with seven cultivars (Table 2). Low minimum temperature was significant only in December and March (Table 3). 

### 3.2. Bud Breaking and Leaf Development

The most noteworthy differences in first phenological phases connected with bud break and leaf development were observed for “American Pillar”, “Lykkefund”, “Polstjårnan”, and “Semiplena”.

**Figure 6:**
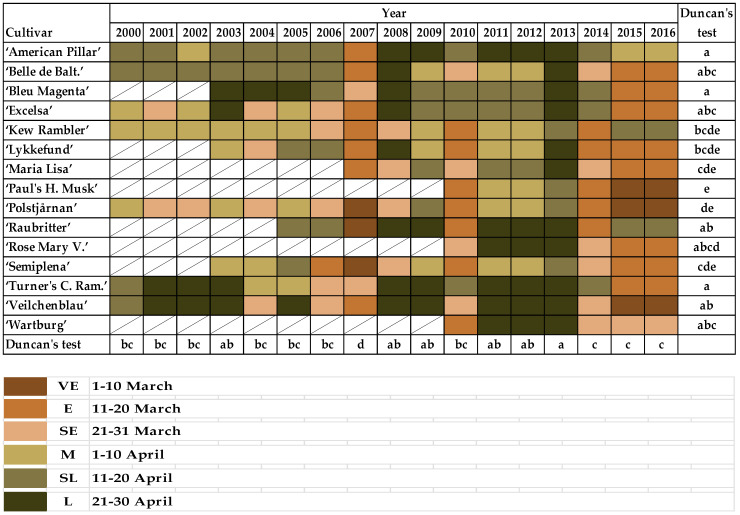
Periods when the buds on unpruned shoots are at the beginning of breaking and the first green leaf tips are visible (BBCH 07) in rambler roses. Different letters indicate significant differences between cultivars and years. The Duncan’s test (α = 0.05) was used.

Most roses began their bud break after 11 April (Figure 6). However, bud break occurred earlier in the last 3 seasons (2013/2014, 2014/2015, and 2015/2016) after mild winters and warm months of March and April. A similar observation was made in 2009/2010 with its warm March and April, in contrast to 2012/2013, with its long winter, frost and snow from December to March, and late but rapid spring in April with temperatures exceeding 25 °C. Bud break was observed the earliest in “Paul’s Himalayan Musk”, then “Kew Rambler”, “Lykkefund”, “Semiplena”, “Polstjårnan”, and occurred the latest in “American Pillar”, “Bleu Magenta”, and “Turner’s Crimson Rambler” (Figure 6).

**Figure 7:**
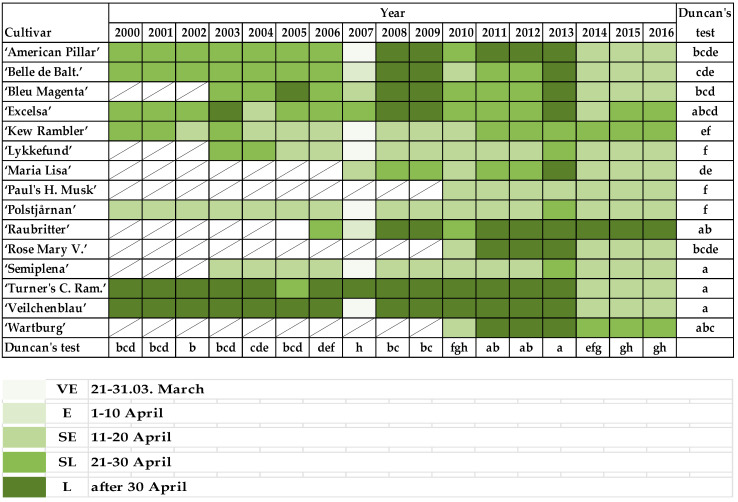
Periods when the first leaf pair has unfolded, not yet at full size, and leaves are light green and/or bronze on unpruned shoots (BBCH 11) of rambler roses. VE—21–31.03. March; E—1–10 April; SE—11–20 April; SL—21–30 April; and L—after 30 April. Different letters indicate significant differences between cultivars and years. The Duncan’s test (α = 0.05) was used.

The first leaf pair unfolded about 10–14 days after bud break every year for most varieties (Figure 7). The earliest first leaf pairs to unfold were observed in “Lykkefund”, “Paul’s Himalayan Musk”, and “Polstjårnan”. “Semiplena”, “Turner’s Crimson Rambler”, and “Veilchenblau” developed their leaves last. The stages of leaf unfolding in climbing roses were similar in the following years (2000–2006 and 2008–2012), and also the determinants of this phenological phase rarely appeared before the 10th of April. An exception was observed in 2007, when leaves appeared on the roses excluding “Bleu Magenta”, “Excelsa”, “Maria Lisa”, “Turner’s Crimson Rambler”, before April 10th, the winter was mild, and the average temperature in March was high (8.1 °C) in relation to other years. This phenological phase also appeared earlier by one or two points in the scale in the years 2014–2016 (Figure 7).

Correlation analysis of the rambler roses for the timing of bud break and leaf folding showed a strict relationship with the average monthly temperature in all cultivars. The decrease of average temperature correlated with late bud break and the early leaf development stage (Table 4 and Table 5).

### 3.3. Flowering

**Figure 8:**
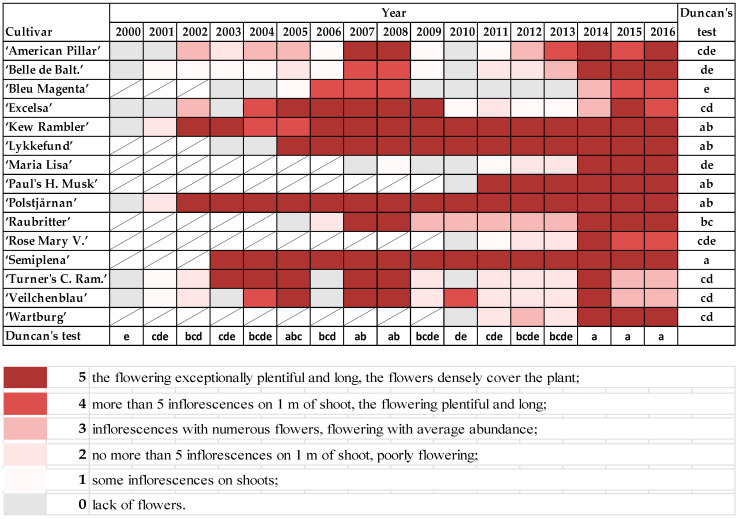
The abundance of flowering in rambler roses on the following scale: 0—lack of flowers; 1—some inflorescences on shoots; 2—no more than 5 inflorescences on 1 m of shoot, but flowering poorly; 3—inflorescences with numerous flowers, but flowering with average abundance; 4—more than 5 inflorescences on 1 m of shoot, plentiful and long flowering; 5—exceptionally plentiful and long flowering, the flowers densely cover the plant. Different letters indicate significant differences in the cultivars and the years. The Duncan’s test (α = 0.05) was used.

The rambler roses started flowering in the first days of June. “Maria Lisa”, “Paul’s Himalayan Musk”, and “Polstjårnan” were the first to start blooming; “Excelsa” and “Rose Mary Viaud” were the last (Figure S4). If the shoots were damaged to the ground (points 6 and 7 on the scale), the low pruned shrubs did not flower, or only a few flowers appeared on old parts of shoots. Exceptionally plentiful and long flowering was observed in “Semiplena” and “Kew Rambler”, “Lykkefund”, “Paul’s Himalayan Musk”, and “Polstjårnan”. Low quality of flowering, especially after frosty winters, were noticed in “Bleu Magenta”, “American Pillar”, “Belle de Baltimore”, “Maria Lisa”, and “Rose Mary Viaud”. Moreover, the flowering was exceptionally plentiful after mild winters (2007, 2008, and 2014−2016) in all plants that were already a few years old (Figure 8).

Correlation analysis of the ramblers for the timing of the start of flowering showed a strict relationship between the average temperature in winter and spring months for all cultivars. A decrease in the average temperature in March correlated with a later start to flowering in “American Pillar”, “Belle de Baltimore”, “Excelsa”, “Polstjårnan”, and “Raubritter”, while a decrease in the average temperature in April was connected with a later start to the flowering of “Kew Rambler” and “Veilchenblau” (Table 6). 

### 3.4. The Correlation between Frost Damage, Early Phenology Stages, and an Abundance of Flowering

The correlation analysis of the rambler roses for all cultivars showed a strict relationship between frost damage, early phenology stages, the timing of the beginning of flowering, and its abundance (Table 7, Table 8 and Table 9). Generally, high frost resistance, a late beginning to the flowering period but with early bud breaking and leaf folding correlated positively with plentiful abundance of flowering. The plants with lower frost resistance (Figure 6) showed a tendency to start bud breaking and leaf development later. These included “American Pillar”, “Belle de Baltimore”, “Bleu Magenta”, “Excelsa”, “Kew Rambler”, “Maria Lisa”, “Paul’s Himalayan Musk”, “Raubritter”, “Rose Mary Viaud”, “Turner’s Crimson Rambler”, “Veilchenblau”, and “Wartburg” (Table 8 and Table 9).

The timing of bud breaking was strictly correlated with the timing of leaf folding. Later timing of the beginning of bud breaking and leaf development contributed to a delay in the start of flowering (Table 7).

However, the results may differ for each individual cultivar (Table 8 and Table 9). Lower frost damage correlated with later flowering in “American Pillar”, “Belle de Baltimore”, “Bleu Magenta”, “Excelsa”, “Paul’s Himalayan Musk”, “Raubritter”, “Veilchenblau”, and “Wartburg”. Earlier bud breaking correlated with later flowering in “American Pillar”, “Bleu Magenta”, “Polstjårnan”, “Semiplena”, and “Veilchenblau” (Table 8 and Table 9).

### 3.5. Growth 

Rose shrubs reached their maximum height in 3 (“Belle de Baltimore”, “Veilchenblau”, and “Wartburg”) to 6 years (“American Pillar”, “Excelsa”, “Maria Lisa”, and “Paul’s Himalayan Musk”) after planting. The necessity of pruning the shoots after severe winters contributed to limited growth and height by the end of the year (Table 10). It was observed that in the years 2014, 2015, and 2016—with an early spring, long autumn, and simultaneously mild winter (Figure S1), and therefore a long growing season—the shrubs were 50–100 cm taller than before, regardless of their age (Table 10).

## 4. Discussion

Changes in the phenology of woody plants [5], roses [9], and crops [40,41] were observed to accompany temporal changes in average temperature and sometimes other weather variables [7]. The difference attributed to changes in atmospheric circulation contributed to the acceleration of several spring phenophases during the years 1951–1998, although these changes were generally greater in Western Europe than in Central or Eastern Europe [44]. This research throughout the years has shown that changes in phenology and overwintering seem to be the effect of climate change resulting from global warming [5,44,45]. This can be easily observed by comparing the plant hardiness zone map published by Heinze and Schreiber in 1984 [46] and the current Plant Map [22]. This tendency was noticed in PAS Botanical Garden CBDC in Powsin (Figure 2, Figures S1 and S3) and Okęcie station (Figure 1). In the research of Zheng et al. [5] most spring and summer phenophases occurred earlier and most autumn and winter phenophases in observed woody species occurred later between 2003 and 2012 than between 1987 and 1996 [5].

The most important criterion that decides on the success of a rose’s cultivation and its ornamental value in a given place is the ability of the shrubs to survive the winter without any influence on their further growth and blooming in present and following seasons. Milder winters in Poland allow for the cultivation of a wider range of plants, some of which would have had no chance of survival even 20–30 years ago [19]. Rambler roses are considered insufficiently resistant to frost and require covering in the Polish climate [21,28]. Similarly to the observed ramblers, many other species, historical groups, and cultivars of roses bloom once per year and start flowering on one-year shoots and older [9], e.g., Spinosissima [47] and Gallica-Hybrids [31]. However, frost damage to the shoots of rambler roses has always resulted in them completely dying out, with visible earlier darkened vascular bundles on shoots or with darkening buds. The shoots, which were initially assessed on a scale of 1 or 2, died out in May. Figure 5 provides the final effect of shoot damage noted in late spring and Table 10 the height that required pruning. The intensely growing shrubs quickly regenerated (Table 10), but nevertheless the pruning reduced blooming in the given year (Figure S4 and Figure 8). However, all varieties, excluding “Bleu Magenta”, flowered in every season between 2011 and 2016, and the last three years were plentiful in flowers (Figure 8). In the research of Pihlajaniemi et al. [15], the differences in winter hardiness between five old shrub varieties of roses were all statistically significant within five sites of observations in North Finland in 1993–1999 [9].

The flowers are a fundamental part of the ornamental value of roses. Overwintering in once-blooming ramblers is especially significant because of their manner of flowering. Flowering was limited, or the shrubs did not bloom at all, in years that followed severe winters (Figures S1 and S4 and Figure 8, Table 7, Table 8 and Table 9), when the shoots were shortened close to the ground or down to the ground itself (at 6–7 on the scale of frost-damage), especially for young shrubs (Table 10). More shoots remained after pruning shrubs that were already a few years old if the frost damage was less than 5 in scale (Figure 5). The flowering would begin a few days later, and the abundance of flowering was always lower in scale in years after substantial winter damage and necessary low pruning, and a few days earlier in years following mild winters and early springs (Figure 8 and Figure S4). Both the longer growing season and higher total monthly temperature in March could be reasons for earlier flowering (Table 7, Table 8 and Table 9). The duration of flowering of Rugosa, Gallica, and Spinosissima in the PAS Botanical Garden in the years 2000–2012 was similar [31,33,47,48]. Additionally, in the research of Pihlajaniemi et al. [9] differences in the ornamental appearance of flowering were statistically significant in the case of six old rose genotypes studied across six years within five sites of observation in North Finland. The mentioned shrub roses are highly frost resistant [9] in comparison to ramblers.

The results of long-term observation also suggest wide adaptability of the observed varieties to the changing and warming climate in the Mazovian Lowland. Warming experiments fail to account for the full magnitude of observed changes in phenology, which suggests that other factors may play important roles here [7]. In the case of this experiment, such factors could include the age and origin of the shrubs. The most studied ramblers originate from Asia, which suggests they may have a lower resistance to frost [25,28]. “Lykkefund” and “Polstjårnan” are highly resistant and did not experience frost damage every year, in contrast to most varieties from the Alba group, e.g., “Celestial”, “Hurdals”, “Maiden’s Blush” [32], Gallica [31], Spinosissima [47], and most old and modern cultivars of Rugosa-Hybrids [33,48], which were all observed in the same climate conditions in the PAS Botanical Garden. It was noted that shrubs of rambler roses that were older in age had a higher tolerance for frost and unfavorable climate conditions than younger specimens, which was also visible at the flowering stage. The warming climate enables the cultivation of less frost resistant plants, however they should nevertheless be protected in the first years of cultivation. It is possible that in the coming years they will be able to survive without protection in microhabitats with suitable conditions [19]. The weather in autumn–winter–spring seasons in the years 2000–2016 was changeable with a tendency for minimal, average, and maximal temperature to increase (Figures S1–S3).

There is only perfunctory information available in the literature on the phenological phases, timing, or duration of the flowering period of old rose cultivars. Throughout the long period of this research, the climate conditions were changing. The timings of phenological phases were different depending on the origin of ramblers but also strictly connected to weather conditions. Similar correlations were observed in other closely connected groups of cultivars, e.g., Spinosissima [47], Rugosa [33,48], Gallica-Hybrids [31], and old shrub genotypes of roses [9]. This relationship was especially relevant in the first phenological phase (BBCH 0) connected with bud break and leaf development (BBCH 1) and with the timing of initial flowering [31,33,47,48]. The results are in line with the view that in temperate climates the timing of phenological phases is particularly dependent on the combination of temperature values [9,20] and photoperiodic courses [49]. Bud break occurred earlier with every next year, except for after frosty winters, when it would take place later. Bud break appeared around a dozen days earlier in later years, especially in 2014–2016, which could be caused by a longer growing season. As Krużel et al. [50] inform, the growing season was an average of 3 days longer in the years 1981–2010 (216–220 days) compared with 1971–2000 in Warsaw, Poland. The growing season in Poland in the years 2001–2009 extended by an average of 8 days as a result of its later termination. It was longer in the northwest of Poland (231–335) and shorter in the east (196–200) [50]. These results are compatible with those of [7], which showed that spring events such as leafing and flowering typically advanced by a median of 4–5 days per degree Celsius [7]. In Poland, accelerated bud break and leaf development after a warmer winter typically exposes the young shoots of rambler roses to night-time spring frost in mid-April and early-May [50,51]. Moreover, research on *Vitis* species showed that changes in phenology caused by climate change could disturb the process of cold hardiness [11]. This process requires dormancy induction in the early winter and is responsible for the maintenance of a dormant state throughout the season, which is crucial to the plants’ survival [11,12].

Moreover, autumn events such as leaf coloring or leaf fall have usually become delayed [7] due to the prolonged growing season [50]. The height of rambler rose shrubs was determined to a large extent by supports, although shrubs of most of the observed cultivars reached their maximum height in autumn independently of the height of spring pruning. The prolonged growing season [50] and growth processes [51] also in rambler roses could disturb the process of hardening before winter dormancy—a period that they require woody plants of the temperate zone [12].

## 5. Conclusions

Ramblers can be cultivated and grow tall in the warmer western parts of Europe, where, because of mild winters, they flower reliably every year [25]. The results confirm that changes in thermal characteristics of the climate of Poland and the associated extension of the meteorological growing season [50,51] and growth season [51] have enabled the introduction of thermophilic plants with higher thermal requirements in cultivation [19,50,51]. Many-year observations of ramblers showed their favorable adaptation to the climate in Poland and the possibility of their wider use in regions with a hitherto cooler climate. However, they have also shown a differentiation in the tolerance of different varieties not only due to frost in winter but also changes in temperature in spring and autumn. These factors had an important influence on the scale of frost damage, growth, and flowering of roses. The average air temperature in autumn-winter-spring correlated strictly with the roses’ early phenological phases.

Rambler roses are a valuable supplement to the available assortment of vines, especially for city greening. Recent years of mild winters and low maintenance made the studied ramblers more useful for a wide range of applications as ornamental plants in parks and rendered them especially preferable for historical garden layout cultivation. This is particularly significant in Central and Eastern Europe, including Poland, where many historical gardens are in poor condition [34].

Currently, rambler roses are not the subject of wide cultivation research, but due to their ornamental merits, which were appreciated in the past, it is worth considering the possibilities for re-establishing their significance. The maintenance of old cultivars, including roses, in cultivation is dependent mostly on resistance to climate conditions.

## Figures and Tables

**Table 1:** The rambler roses observed in the National Collection of Rose Cultivars of PAS Botanical Garden CBDC in Powsin.

Cultivar	Introduction	Group/Origin	Number of Shrubs	Year of Planting	Height of Support (m)
“American Pillar”	W. van Fleet, 1902	Hybrid Wichurana	6	1998	2.0
“Belle de Baltimore”	S.&J. Feast, 1843	Hybrid Setigera	6	1998	no
“Bleu Magenta”	G.R. du Val de Loire, 1933	Hybrid Multiflora	5	2002	2.0
“Excelsa”	M.H. Walsh, 1908	Hybrid Wichurana	8	1998	2.0
“Kew Rambler”	Unknown, 1912	*Rosa soulieana* Crép.	5	1998	2.4
“Lykkefund”	A. Olsen, 1930	*R. helenae* Rehder and Wilson	3	2003	2.0
“Maria Lisa”	B. Alfons, 1925	Hybrid Multiflora	5	2006	2.4
“Paul’s Himalayan Musk”	G. Paul, 1916	*Rosa brunnonii* Lindl.	3	2009	2.4
“Polstjårnan”	Wasastjerna, 1937	*R. beggeriana* Schrenk ex Fisch. and C.A.Mey.	3	1998	no
“Raubritter”	W.J.H. Kordes II, 1936	Hybrid Macrantha	5	2005	1.5
“Rose Mary Viaud”	M. Igoult, 1924	Hybrid Multiflora	5	2009	2.0
“Semiplena”	Unknown	*R. helenae* Rehder and Wilson	3	2003	2.0
“Turner’s Crimson Rambler”	Unknown, before 1893	Hybrid Multiflora	3	1998	2.4
“Veilchenblau”	J.Ch. Schmidt, 1909	Hybrid Multiflora	9	1998	2.4
“Wartburg”	H. Kiese, 1910	Hybrid Multiflora	9	2009	2.4

**Table 2:** Correlations between average monthly air temperature (2005/2006, 2009/2010, and 2015/2016) and frost damage of rambler roses.

Cultivar	SD	Month						
		October	November	December	January	February	March	April
“American Pillar”	0.58	−0.763 **	−0.104	−0.388	−0.998 ***	−0.981 ***	0.455	−0.106
“Belle de Baltimore”	2.89	−0.763 **	−0.104	−0.388	−0.998 ***	−0.981 ***	0.455	−0.106
“Bleu Magenta”	1.53	−0.932 ***	0.227	−0.668 **	−0.940 ***	−0.862 **	0.722 **	−0.426
“Excelsa”	1.00	−0.984 ***	0.407	−0.797 **	−0.859 **	−0.751 **	0.840 **	−0.589 *
“Kew Rambler”	1.73	−0.179	0.914 ***	−0.604 **	0.512 *	0.660 **	0.543 *	−0.808 **
“Lykkefund”	2.31	−0.941 ***	0.809 **	−0.992 ***	−0.488	−0.320	0.999 ***	−0.914 ***
“Maria Lisa”	1.15	−0.763 **	−0.104	−0.388	−0.998 ***	−0.981 ***	0.455	−0.106
“Paul’s Him. Musk”	1.15	−0.763 **	−0.104	−0.388	−0.998 ***	−0.981 ***	0.455	−0.106
“Polstjårnan”	0.58	0.179	−0.914 ***	0.604 **	−0.512 *	−0.660 **	−0.543 *	0.808 **
“Raubritter”	2.65	−0.871 **	0.085	−0.555 *	−0.979 ***	−0.926 ***	0.616 **	−0.292
“Rose Mary Viaud”	1.15	−0.763 **	−0.104	−0.388	−0.998 ***	−0.981 ***	0.455	−0.106
“Semiplena”	2.31	−0.941 ***	0.809 **	-0.992 ***	−0.488	−0.320	0.999 ***	−0.914 ***
“Turner’s Crim. Ram.”	1.15	−0.763 **	−0.104	−0.388	−0.998 ***	−0.981 ***	0.455	−0.106
“Veilchenblau”	1.15	−0.763 **	-0.104	−0.388	−0.998 ***	−0.981 ***	0.455	−0.106
“Wartburg”	0.58	0.179	−0.914 ***	0.604 **	−0.512 *	−0.660 **	−0.543 *	0.808 **

Marked correlations are significant at *p* < 0.05. Correlation significance: * 0.500–0.599—restrained; ** 0.600–0.899—high; *** >0.9—very high.

**Table 3:** Part of matrices concerning effect correlations between minimum monthly air temperatures (2005/2006, 2009/2010, and 2015/2016) and frost damage of rambler roses.

Cultivar	SD	Month						
		October	November	December	January	February	March	April
“American Pillar”	2.11	−0.028	−0.161	−0.562 *	−0.525 *	−0.242	−0.659 **	−0.033
“Belle de Baltimore”	2.55	0.221	−0.038	−0.506 *	−0.481	−0.501	−0.684 **	−0.328
“Bleu Magenta”	2.32	0.137	−0.155	−0.502 *	−0.445	−0.263	−0.676 **	0.085
“Excelsa”	2.89	0.079	0.114	−0.501 *	−0.590 *	−0.341	−0.448	−0.111
“Kew Rambler”	2.14	−0.027	0.080	−0.472	−0.328	−0.274	−0.257	0.249
“Lykkefund”	1.21	−0.118	0.262	−0.419	−0.388	0.055	−0.198	0.248
“Maria Lisa”	2.57	0.066	−0.025	−0.448	−0.416	−0.386	−0.694 **	−0.269
“Paul’s Him. Musk”	2.46	−0.034	−0.027	−0.562 *	−0.320	−0.277	−0.302	0.344
“Polstjårnan”	2.45	−0.064	−0.162	0.143	0.049	0.234	−0.088	−0.071
“Raubritter”	2.38	−0.048	−0.162	0.089	0.005	0.181	−0.159	−0.108
“Rose Mary Viaud”	2.27	−0.074	−0.114	−0.542 *	−0.377	−0.305	−0.749 **	−0.180
“Semiplena”	1.21	−0.118	0.262	−0.419	−0.388	0.055	−0.198	0.248
“Turner’s Crim. Ram.”	2.27	−0.074	−0.114	−0.542 *	−0.377	−0.305	−0.749 **	−0.180
“Veilchenblau”	2.36	−0.068	−0.168	0.089	−0.003	0.207	−0.147	−0.086
“Wartburg”	2.15	−0.079	−0.250	−0.465	−0.328	−0.236	−0.687 **	−0.225

Marked correlations are significant at *p* < 0.05. Correlation significance: * 0.500–0.599—restrained; ** 0.600–0.899—high.

**Table 4:** Part of the matrices of effect correlations between average monthly air temperature (2005/2006, 2009/2010, and 2015/2016) and periods when the buds on unpruned shoots are at the beginning of breaking and the first green leaf tips are visible (BBCH 07) in rambler roses.

Cultivar	SD	Month						
		October	November	December	January	February	March	April
“American Pillar”	5.77	−0.763 **	−0.104	−0.388	−0.998 ***	−0.981 ***	0.455	−0.106
“Belle de Baltimore”	15.28	−0.153	−0.730 **	0.310	−0.765 **	−0.870 ***	−0.238	0.571 *
“Bleu Magenta”	17.32	−0.763 **	−0.104	−0.388	−0.998 ***	−0.981 ***	0.455	−0.106
“Excelsa”	15.28	−0.998	0.572 *	−0.897 **	−0.747 **	−0.613 *	0.927 ***	−0.731 **
“Kew Rambler”	15.28	0.932 ***	−0.227	0.668 *	0.940 ***	0.862 **	−0.722 **	0.426
“Lykkefund”	17.32	0.179	−0.914 ***	0.604 *	−0.512 *	−0.660 *	−0.543 *	0.808 **
“Maria Lisa”	5.00	−0.984 ***	0.407	−0.797 **	−0.859 **	−0.751 **	0.840 **	−0.589 *
“Paul’s Him. Musk”	5.01	−0.993 ***	0.459	−0.830 **	−0.828 **	−0.712 **	0.869 **	−0.635 *
“Polstjårnan”	10.00	−0.337	−0.588 *	0.125	−0.873 **	−0.947 ***	−0.051	0.405
“Raubritter”	17.32	0.941 ***	−0.809 **	0.992 ***	0.488	0.320	−0.999 ***	0.914 ***
“Rose Mary Viaud”	5.00	−0.984 ***	0.407	−0.797 **	−0.859 **	−0.751 **	0.840 **	−0.589 *
“Semiplena”	0.58	−0.76388	−0.104	−0.388	−0.998 ***	−0.981 ***	0.455	−0.106
“Turner’s Crim. Ram.”	15.28	−0.998 ***	0.572 *	−0.897 **	−0.747 **	−0.613 *	0.927 ***	−0.731 **
“Veilchenblau”	11.55	−0.763 **	−0.104	−0.388	−0.998 ***	−0.981 ***	0.455	−0.106
“Wartburg”	5.00	0.984 ***	−0.407	0.797 **	0.859 **	0.751 **	−0.840 **	0.589 *

Marked correlations are significant at *p* < 0.05. Correlation significance: * 0.500–0.699—restrained; ** 0.700–0.899—high; *** >0.900—very high.

**Table 5:** Part of matrices of effect correlations between average monthly air temperature (2005/2006, 2009/2010, and 2015/2016) and timing of the first leaf pair unfolding, not yet at full size, and light green and/or bronze (BBCH 11) in color in rambler roses.

Cultivar	SD	Month						
		October	November	December	January	February	March	April
“American Pillar”	5.77	−0.763 **	−0.104	−0.388	−0.998 ***	−0.981 ***	0.455	−0.106
“Belle de Balt.”	5.77	0.179	−0.914 ***	0.604 *	−0.512 *	−0.660 *	−0.543 *	0.808 **
“Bleu Magenta”	5.77	−0.763 **	−0.104	−0.388	−0.998 ***	−0.981 ***	0.455	−0.106
“Excelsa”	0.58	0.763 **	0.104	0.388	0.998 ***	0.981 ***	−0.455	0.106
“Kew Rambler”	5.77	0.763 **	0.104	0.388	0.998 ***	0.981 ***	−0.455	0.106
“Lykkefund”	0.58	−0.763 **	−0.104	−0.388	−0.998 ***	−0.981 ***	0.455	−0.106
“Maria Lisa”	0.58	−0.763 **	−0.104	−0.388	−0.998 ***	−0.981 ***	0.455	−0.106
“Paul’s Him. Musk”	0.58	0.179	−0.914 ***	0.604 *	−0.512 *	−0.660 *	−0.543 *	0.808 **
“Polstjårnan”	0.58	−0.763 **	−0.104	−0.388	−0.998 ***	−0.981 ***	0.455	−0.106
“Raubritter”	5.77	0.763 **	0.104	0.388	0.998 ***	0.981 ***	−0.455	0.106
“Rose Mary Viaud”	0.58	−0.763 **	−0.104	−0.388	−0.998 ***	−0.981 ***	0.455	−0.106
“Semiplena”	0.58	−0.763 **	−0.104	−0.388	−0.998 ***	−0.981 ***	0.455	−0.106
“Turner’s Crim. Ram.”	10.00	−0.984 ***	0.407	−0.797 **	−0.859 **	−0.751 **	0.840 **	−0.589 *
“Veilchenblau”	11.55	−0.763 **	−0.104	−0.388	−0.998 ***	−0.981 ***	0.455	−0.106
“Wartburg”	5.00	0.984 ***	−0.407	0.797 **	0.859 **	0.751 **	−0.840 **	0.589 *

Marked correlations are significant at *p* < 0.05. Correlation significance: * 0.500−0.699—restrained; ** 0.700–0.899—high; *** >0.900—very high.

**Table 6:** The part of matrices of effect correlations between average monthly air temperature (2006, 2010, and 2016) and the start of the flowering period (BBCH 60 601) in rambler roses.

Cultivar	SD	Month						
		October	November	December	January	February	March	April
“American Pillar”	9.87	0.971 ***	−0.745 **	0.974 ***	0.574 *	0.414	−0.988 ***	0.868 ***
“Belle de Baltimore”	7.64	0.779 **	−0.957 ***	0.978 ***	0.176	−0.008	−0.960 ***	0.996 ***
“Bleu Magenta”	9.81	0.763 **	0.104	0.388	0.998 ***	0.981 ***	−0.455	0.106
“Excelsa”	11.55	0.179	−0.914 ***	0.604 *	−0.512 *	−0.661 *	−0.543 *	0.808 **
“Kew Rambler”	2.52	−0.555 *	0.998 ***	−0.871 ***	0.128	0.308	0.832 **	−0.976 ***
“Lykkefund”	5.51	−0.701 **	−0.194	−0.303	−0.997 ***	−0.995 ***	0.373	−0.015
“Maria Lisa”	0.58	0.763 **	0.104	0.388	0.998 ***	0.981 ***	−0.455	0.106
“Paul’s Him. Musk”	4.04	0.763 **	0.104	0.388	0.998 ***	0.981 ***	−0.455	0.106
“Polstjårnan”	7.55	0.555 *	−0.998 ***	0.871 **	−0.128	−0.308	−0.832 **	0.976 ***
“Raubritter”	6.51	0.612 *	−0.998 ***	0.903 ***	−0.058	−0.240	−0.869 **	0.989 ***
“Rose Mary Viaud”	8.66	0.763 **	0.104	0.388	0.998 ***	0.981 ***	−0.455	0.106
“Semiplena”	0.58	−0.763 **	−0.104	−0.388	−0.998 ***	−0.981 ***	0.455	−0.106
“Turner’s Crim. Ram.”	5.77	0.763 **	0.104	0.388	0.998 ***	0.981 ***	−0.455	0.106
“Veilchenblau”	2.52	−0.730 **	0.976 ***	−0.960 ***	−0.101	0.083	0.937 ***	−0.998 ***
“Wartburg”	0.58	0.763 **	0.104	0.388	0.998 ***	0.981 ***	−0.455	0.106

Marked correlations are significant at *p* < 0.05. Correlation significance: * 0.500–0.699—restrained; ** 0.700–0.899—high; *** >0.900—very high.

**Table 7:** Correlation matrices between frost damage, early phenology stages, beginning of flowering, and its abundance in rambler roses for all cultivars taken together.

Variable	Bud Break	Leaf Development	Frost Damage	Beginning of Flowering	Abundance of Flowering
For all Cultivars					
Leaf Development	0.700 ****	1.000			
Frost Damage	0.380 **	0.491 **	1.000		
Beginning of Flowering	0.118 *	0.162 *	0.058	1.000	
Abundance of Flowering	−0.392 **	−0.388 **	−0.560 ***	0.283 *	1.000

Note: Marked correlations are significant at α= 0.01. Correlation significance: * 0.100–0.299—low; ** 0.300–0.499—restrained; *** 0.500–0.699—high; **** 0.700—0.899—very highly.

**Table 8:** Correlation matrices between frost damage, early phenology stages, beginning of flowering, and its abundance for “American Pillar”, “Belle Herminie”, “Bleu Magenta”, “Excelsa”, “Kew Rambler”, “Lykkefund”, “Maria Lisa”, and “Paul’s Himalayan Musk”.

**Variable**	**Bud Break**	**Leaf Development**	**Frost Damage**	**Beginning of Flowering**	**Abundance of Flowering**
**“American Pillar”**					
Leaf Development	0.941 ****	1.000			
Frost Damage	0.390 **	0.494 **	1.000		
Beginning of Flowering	−0.119 *	−0.060	−0.153 *	1.000	
Abundance of Flowering	−0.326 **	−0.389 **	−0.651 ***	0.531 ***	1.000
**“Belle de Baltimore”**					
Leaf Development	0.842 ****	1.000			
Frost Damage	0.428 **	0.498 **	1.000		
Beginning of Flowering	−0.032	−0.248 *	−0.298 *	1.000	
Abundance of Flowering	−0.505 ***	−0.361 **	−0.793 ****	0.379 **	1.000
**“Bleu Magenta”**					
Leaf Development	0.758 ****	1.000			
Frost Damage	0.154 *	0.355 **	1.000		
Beginning of Flowering	−0.420 **	−0.141 *	−0.594 ***	1.000	
Abundance of Flowering	−0.623 ***	−0.352 **	−0.446 **	0.662 ***	1.000
**“Excelsa”**					
Leaf Development	0.500 ***	1.000			
Frost Damage	0.382 **	0.349 **	1.000		
Beginning of Flowering	0.038	−0.220 *	−0.104 *	1.000	
Abundance of Flowering	−0.411 **	−0.091	−0.552 ***	0.608 ***	1.000
**“Kew Rambler”**					
Leaf Development	0.563 ***	1.000			
Frost Damage	0.170 *	0.143 *	1.000		
Beginning of Flowering	−0.045	−0.084	0.374 **	1.000	
Abundance of Flowering	−0.136 *	−0.222 *	0.380 **	0.776 ****	1.000
**“Lykkefund”**					
Leaf Development	0.377 **	1.000			
Frost Damage	−0.315 **	−0.028	1.000		
Beginning of Flowering	0.085	−0.042	0.440 **	1.000	
Abundance of Flowering	0.040	−0.539 ***	0.113 *	0.473	1.000
**“Maria Lisa”**					
Leaf Development	0.887 ****	1.000			
Frost Damage	0.624 ***	0.435 **	1.000		
Beginning of Flowering	0.496 **	0.583 ***	0.039	1.000	
Abundance of Flowering	−0.367 **	−0.343 **	−0.347 **	0.324 **	1.000
**“Paul’s Himalayan Musk”**					
Leaf Development	−0.269 *	1.000			
Frost Damage	0.426 **	−0.551 ***	1.000		
Beginning of Flowering	0.132 *	0.452 **	−0.492 **	1.000	
Abundance of Flowering	0.196 *	0.340 **	−0.490 **	0.870 ****	1.000

Note: Marked correlations are significant at α = 0.01. Correlation significance: * 0.100–0.299—low; ** 0.300–0.499—restrained; *** 0.500–0.699—high; **** 0.700—0.899—very highly.

**Table 9:** Correlation matrices between frost damage, early phenology stages, the beginning of flowering, and its abundance in rambler roses: “Polstjårnan”, “Raubritter”, “Rose Mary Viaud”, “Semiplena”, “Turner’s Crimson Rambler”, “Veilchenblau”, and “Wartburg”.

**Variable**	**Bud Break**	**Leaf Development**	**Frost Damage**	**Beginning of Flowering**	**Abundance of Flowering**
**“Polstjårnan** **”**					
Leaf Development	0.525 ***	1.000			
Frost Damage	Nd	Nd	1.000		
Beginning of Flowering	−0.170 *	−0.034	Nd	1.000	
Abundance of Flowering	−0.159 *	−0.039	Nd	0.289 *	1.000
**“Raubritter”**					
Leaf Development	0.499 **	1.000			
Frost Damage	0.320 **	−0.046	1.000		
Beginning of Flowering	−0.049	0.096	−0.334 **	1.000	
Abundance of Flowering	−0.301 **	0.400 **	−0.799 ****	0.477 **	1.000
**“Rose Mary Viaud”**					
Leaf Development	0.977 *****	1.000			
Frost Damage	0.599 ***	0.636 ***	1.000		
Beginning of Flowering	0.564 ***	0.663 ***	0.001	1.000	
Abundance of Flowering	−0.528 ***	−0.467 **	−0.869 ****	0.339 **	1.000
**“Semiplena”**					
Leaf Development	0.620 ***	1.000			
Frost Damage	−0.279 *	0.033	1.000		
Beginning of Flowering	−0.283 *	0.326 **	0.349 **	1.000	
Abundance of Flowering	Nd	Nd	Nd	Nd	1.000
**“Turner’s C. Rambler”**					
Leaf Development	0.603 ***	1.000			
Frost Damage	0.219 *	0.371	1.000		
Beginning of Flowering	0.177 *	−0.298 *	−0.084	1.000	
Abundance of Flowering	−0.222 *	0.070	−0.487 **	0.616 ***	1.000
**“Veilchenblau”**					
Leaf Development	0.716 ****	1.000			
Frost Damage	0.259 *	0.606 ***	1.000		
Beginning of Flowering	−0.391 **	0.214 *	−0.294 *	1.000	
Abundance of Flowering	0.369 **	−0.455 **	−0.710 ****	0.345 **	1.000
**“Wartburg”**					
Leaf Development	0.959 *****	1.000			
Frost Damage	0.459 **	0.344 **	1.000		
Beginning of Flowering	0.603 ***	0.757 ****	−0.112 *	1.000	
Abundance of Flowering	−0.200 *	0.081	−0.458 **	0.494 **	1.000

Note: Marked correlations are significant at α = 0.01. Correlation significance: * 0.100–0.299—low; ** 0.300–0.499—restrained; *** 0.500–0.699—high; **** 0.700–0.899—very highly; ***** ≥0.900—almost full. Nd—The variable takes a constant value.

**Table 10:** The height (cm) of shrubs of rambler roses after spring pruning (S) and at the end of October (A).

Cultivar	Term	Year																
		2000	2001	2002	2003	2004	2005	2006	2007	2008	2009	2010	2011	2012	2013	2014	2015	2016
“American Pillar”	S	60	100	100	60	150	200	200	300	300	150	200	150	150	200	250	250	250
	A	60	150	150	200	250	250	300	300	300	200	200	150	250	250	250	250	250
“Belle de Baltimore”	S	100	100	100	100	100	100	100	150	200	60	60	60	100	150	150	150	200
	A	150	150	150	150	150	150	150	200	200	80	150	150	150	150	150	200	200
“Bleu Magenta”	S	-	-	-	60	60	60	100	100	150	30	30	30	30	100	150	150	150
	A	-	-	-	100	100	100	100	150	150	80	150	80	150	150	150	150	150
“Excelsa”	S	30	30	100	30	30	150	50	300	300	60	60	60	60	60	300	300	250
	A	60	150	150	150	200	200	300	300	300	200	200	250	300	300	300	300	300
“Kew Rambler”	S	60	200	200	200	200	200	100	250	250	250	250	300	300	300	300	350	350
	A	200	200	200	200	200	200	200	250	250	300	300	300	300	300	300	350	350
“Lykkefund”	S	-	-	-	30	100	200	200	250	250	250	250	300	300	300	300	300	300
	A	-	-	-	100	200	200	200	250	250	300	300	300	300	300	300	300	300
“Maria Lisa”	S	-	-	-	-	-	-	-	30	100	30	100	150	200	200	300	300	250
	A	-	-	-	-	-	-	-	100	200	100	150	200	300	300	300	300	300
“Paul’s Him. Musk”	S	-	-	-	-	-	-	-	-	-	-	50	150	200	300	500	500	500
	A	-	-	-	-	-	-	-	-	-	-	150	150	200	300	500	500	500
“Polstjårnan”	S	100	250	250	300	300	300	300	300	300	300	300	300	300	300	300	300	300
	A	100	250	250	300	300	300	300	300	300	300	300	300	300	300	300	300	300
“Raubritter”	S	-	-	-	-	-	40	120	200	200	170	200	300	300	300	300	300	250
	A	-	-	-	-	-	100	150	200	200	170	200	300	300	300	300	300	250
“Rose Mary Viaud”	S	-	-	-	-	-	-	-	-	-	-	40	80	80	80	200	180	180
	A	-	-	-	-	-	-	-	-	-	-	80	80	150	150	200	200	200
“Semiplena”	S	-	-	-	100	150	150	200	250	250	250	250	250	300	300	300	300	300
	A	-	-	-	100	150	150	200	250	250	250	250	250	300	300	300	300	300
“Turner’s Crim. Ram.”	S	30	70	100	100	100	100	20	200	200	20	20	20	60	60	200	200	200
	A	70	100	150	150	200	200	200	200	200	150	200	200	200	200	250	250	250
“Veilchenblau”	S	30	70	100	20	100	100	20	200	200	20	20	150	150	150	200	200	200
	A	60	150	150	200	200	200	200	200	200	200	200	200	200	200	200	300	300
“Wartburg”	S	-	-	-	-	-	-	-	-	-	-	30	30	150	200	250	200	200
	A	-	-	-	-	-	-	-	-	-	-	100	150	250	250	300	300	300

## Supplementary Materials

The following are available online at https://www.mdpi.com/2223-7747/10/3/457/s1, Figure S1: The minimal (blue line), average twenty-four (black line) and maximal (red line) air temperature (°C) from October to April in the autumn-winter seasons of 2002/2003, Figure S2: The total monthly precipitation (mm) and month mean in the years 2000–2016, measured in the PAS Botanical Garden CBDC in Powsin, Figure S3: The total monthly average air temperatures (°C) in the years 2000–2016, measured in the PAS Botanical Garden CBDC in Powsin, Figure S4: Data concerning the start of flowering, full flowering and its end in rambler roses in the years 2000–2016. The 1st day of flowering scale begins at 25 May. Different small letters indicate significant differences between cultivars and capital letters between years, according to the timing of the start of flowering. The Duncan’s test (α = 0.05) was used.
