# Supplementary material for: Response of Rambler Roses to Changing Climate Conditions in Urbanized Areas of the European Lowlands
Source: Plants (Basel). 2021 Feb 28;10(3):457. doi: 10.3390/plants10030457 (PMC7997323; doi:10.3390/plants10030457)
Supplement: Supplementary file 1 [file plants-10-00457-s001.zip › Supplementary file.docx]

*Article*

**Response of Rambler Roses to Changing Climate Conditions** **in Urbanized Areas of the European Lowlands**

**Marta Joanna Monder**

Polish Academy of Sciences Botanical Garden – Center for Biological Diversity Conservation in Powsin, Department of Dendrological Collections; Prawdziwka 2, 02-973 Warsaw, Poland;

**Correspondenc*e: m.monder@obpan.pl

**b**

**a**


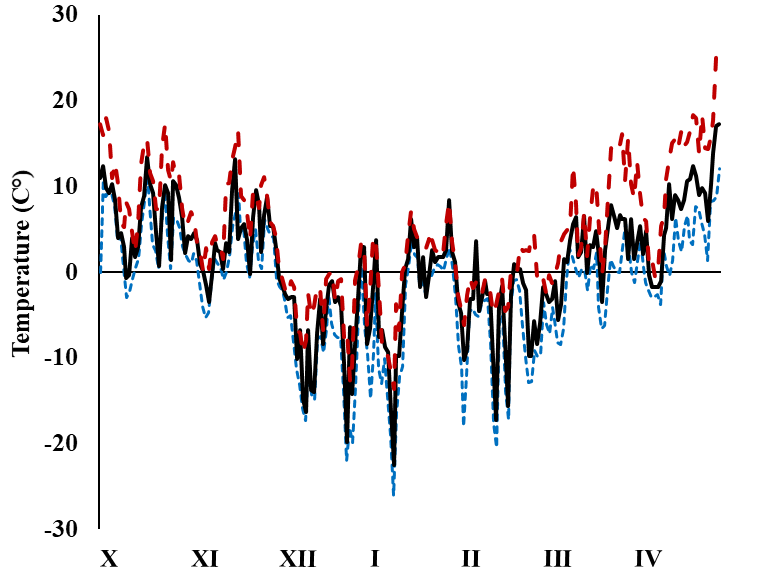

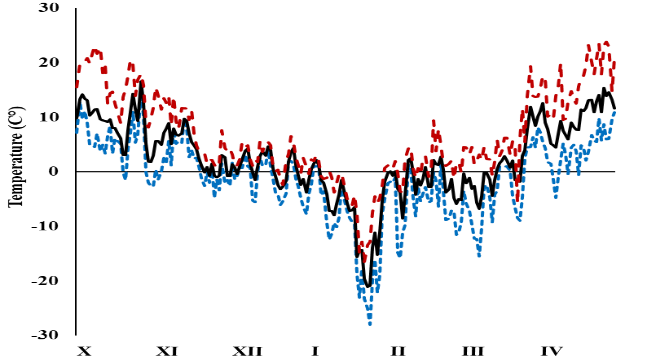


**d**

**c**


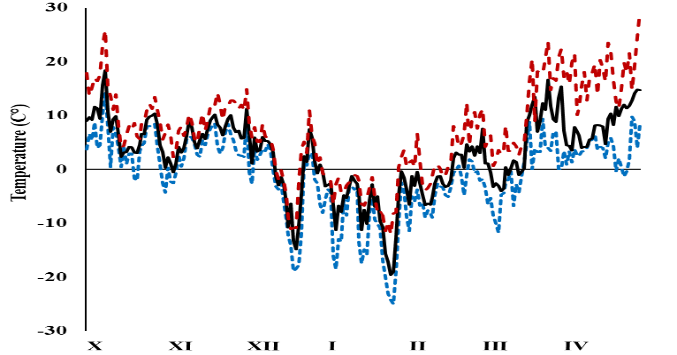

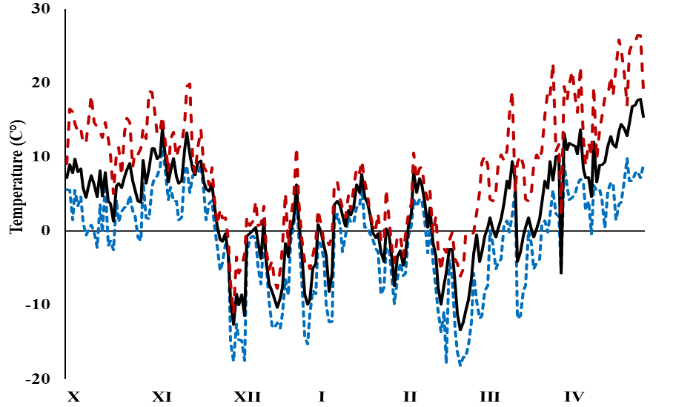


**f**

**e**


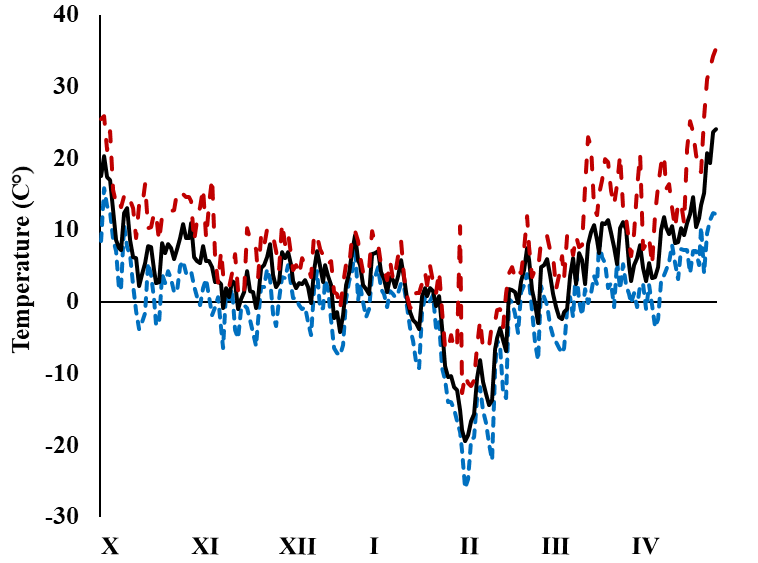

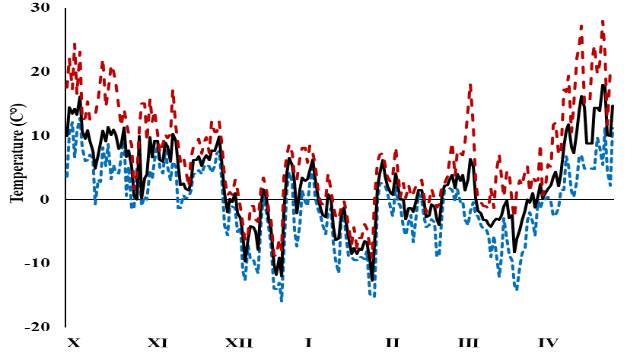


**h**

**g**


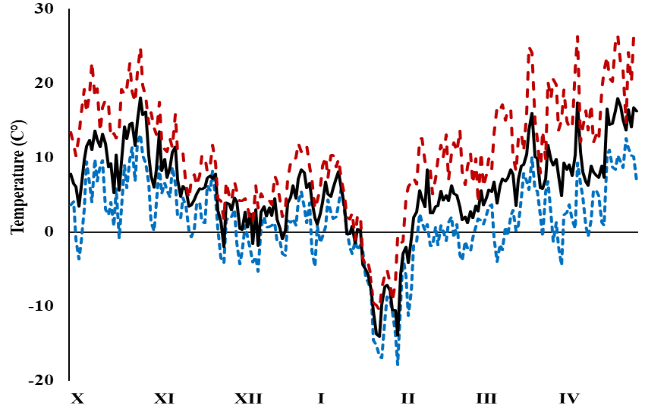

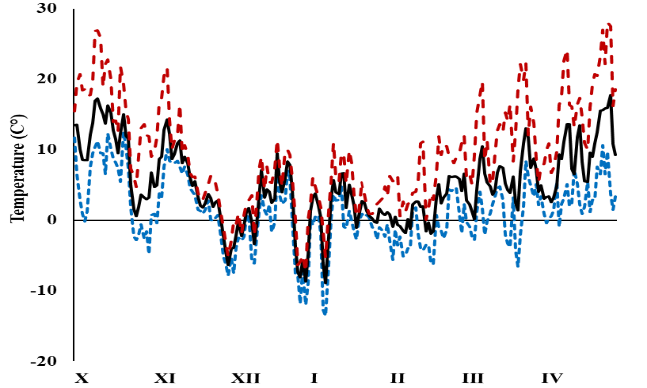


**i**

**
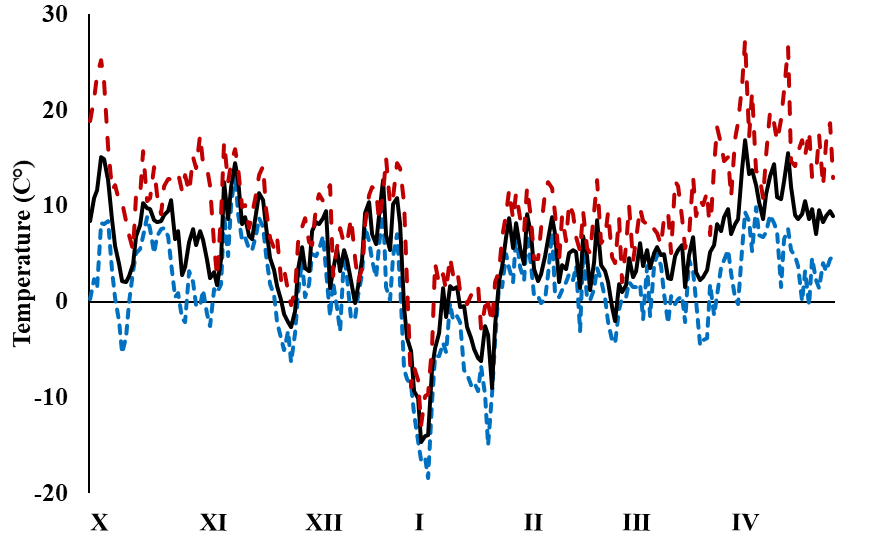
**

**Maximal temperature**

**Average twenty-four temperature**

**Minimal temperature**

**Figure S1**. The minimal (blue line), average twenty-four (black line) and maximal (red line) air temperature [°C] from October to April in the autumn-winter seasons of 2002/2003 (a), 2005/2006 (b) and 2009/2010 (c), 2010/2011 (d), 2011/2012 (e) and 2012/2013 (f), 2013/2014 (g), 2014/2015 (h).


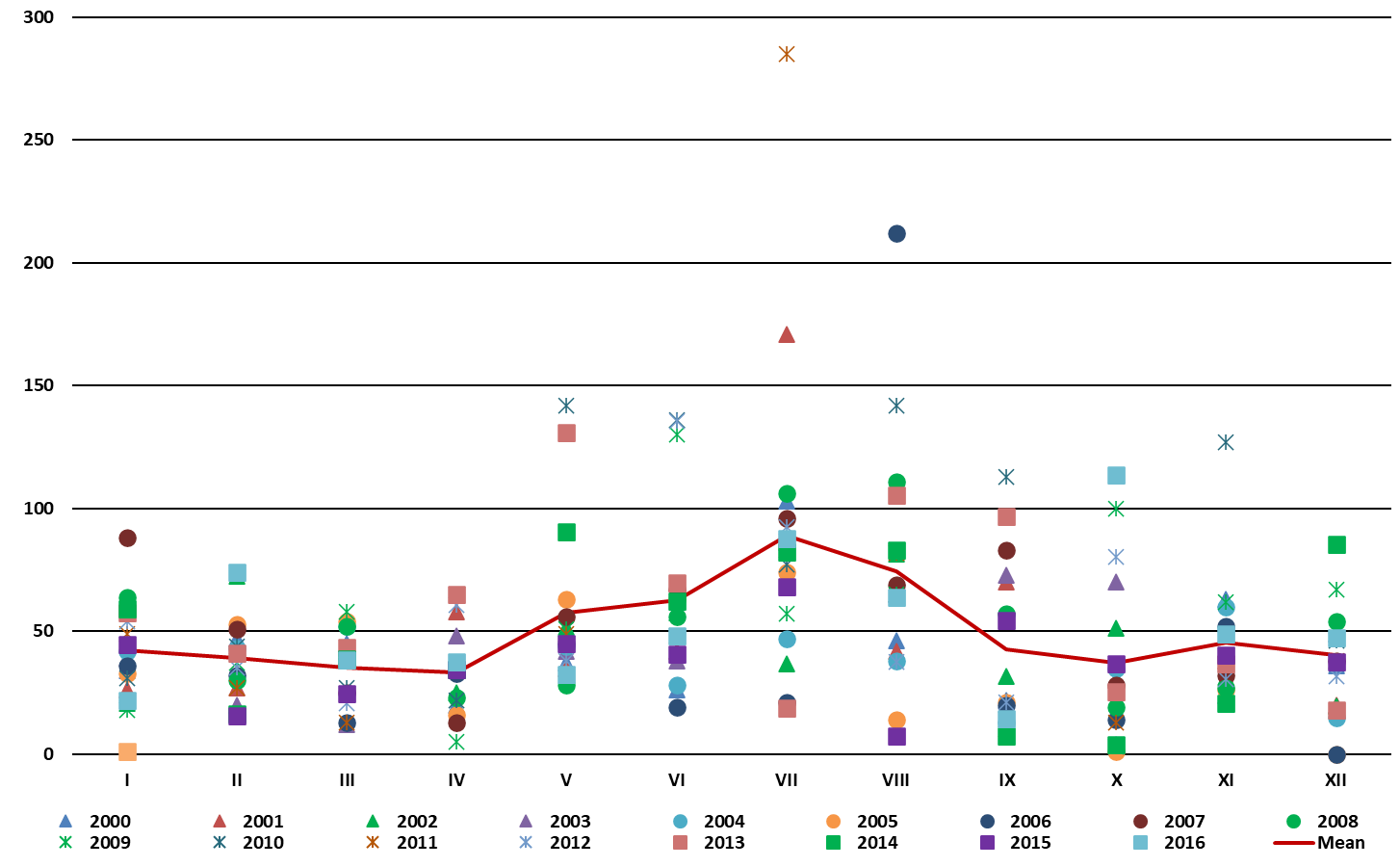


**Figure S2**. The total monthly precipitation [mm] and month mean in the years 2000-2016, measured in the PAS Botanical Garden CBDC in Powsin.


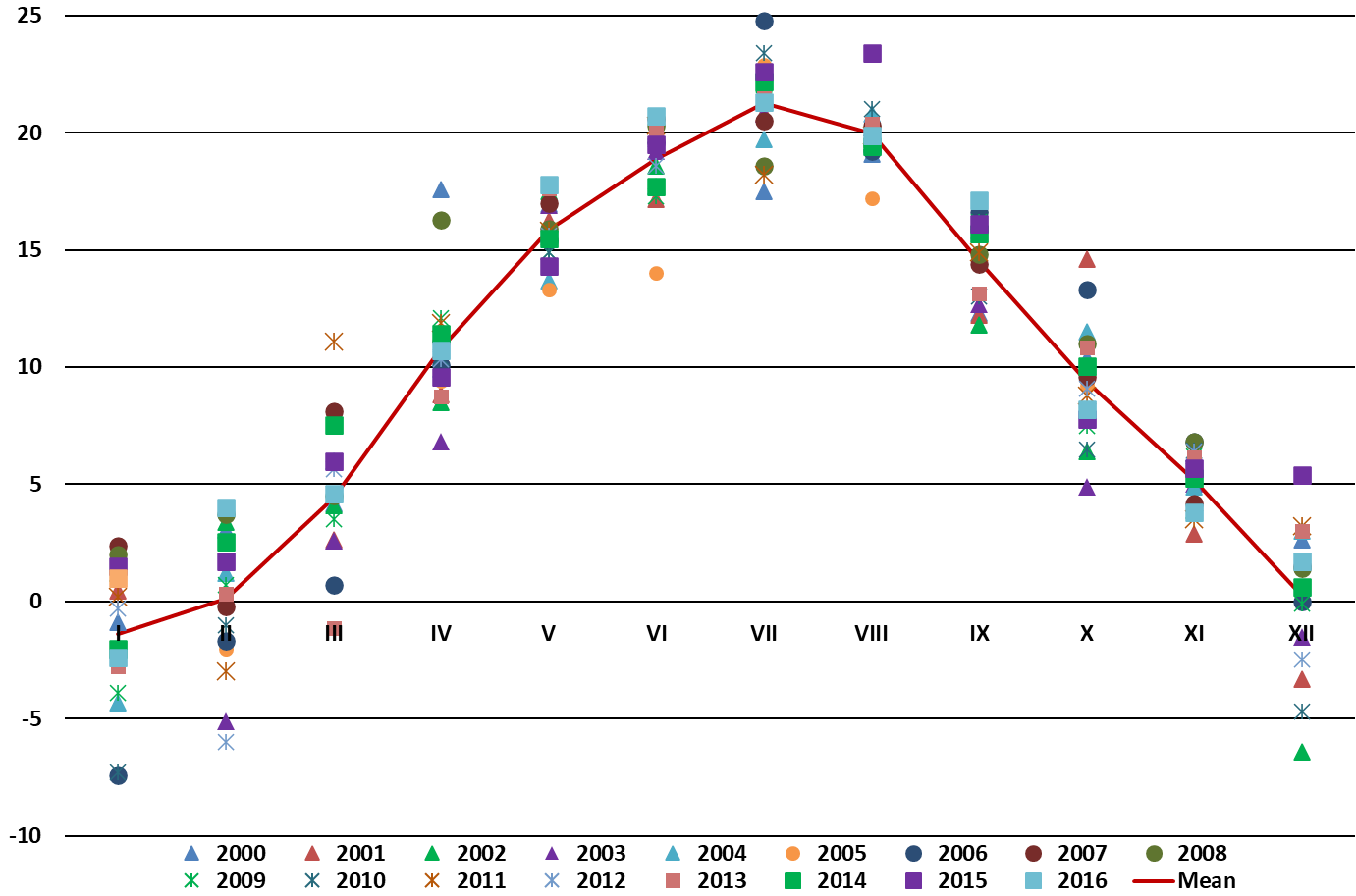


**Figure S3**. The total monthly average air temperatures [°C] in the years 2000-2016, measured in the PAS Botanical Garden CBDC in Powsin.
