# Supplementary material for: Response of Rambler Roses to Changing Climate Conditions in Urbanized Areas of the European Lowlands
Source: Plants (Basel). 2021 Feb 28;10(3):457. doi: 10.3390/plants10030457 (PMC7997323; doi:10.3390/plants10030457)
Supplement: Supplementary file 1 [file plants-10-00457-s001.zip › Figure S4.pptx]

## Slide 1
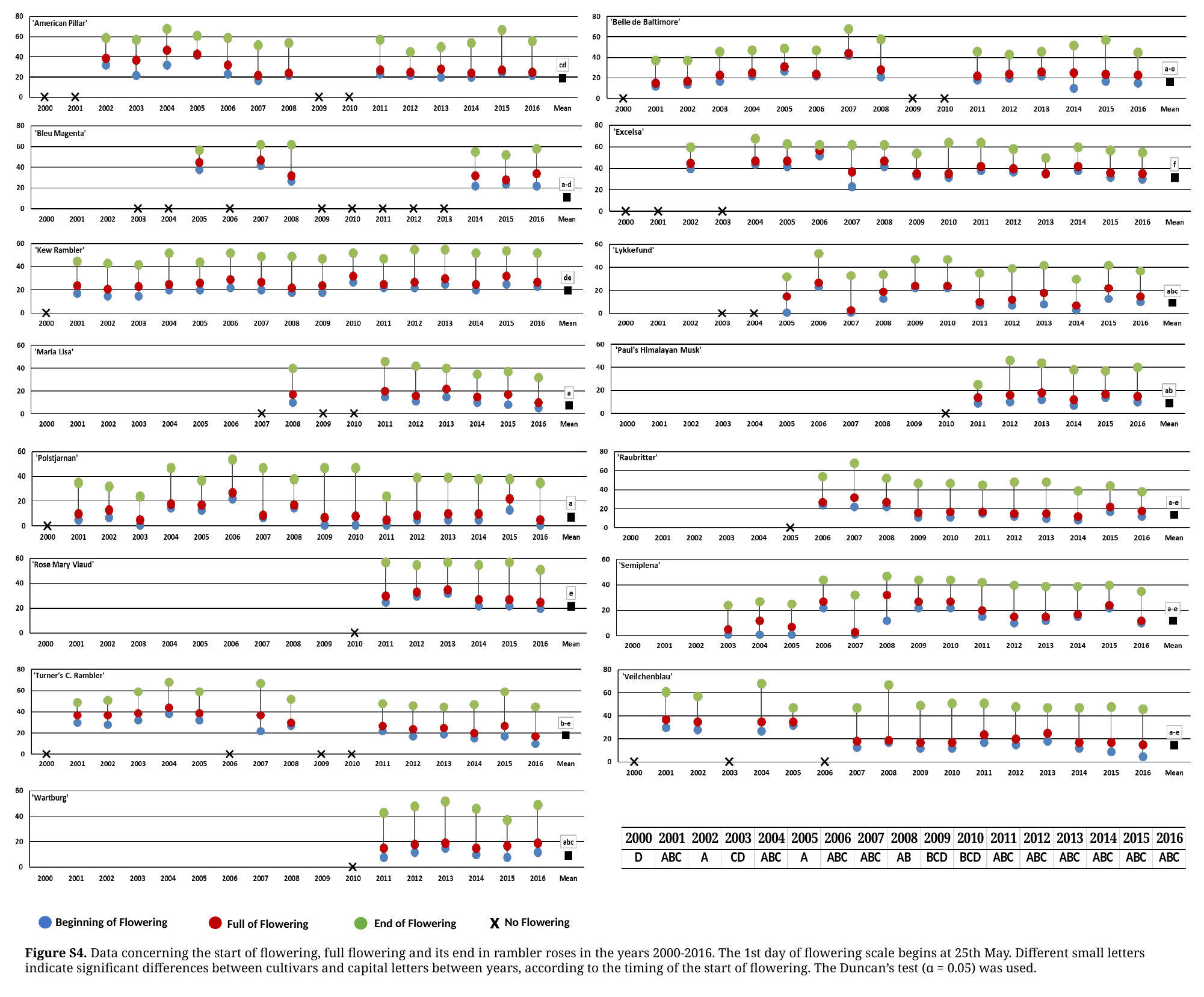

x
Beginning of Flowering
No Flowering
End of Flowering
Full of Flowering
Figure S4. Data concerning the start of flowering, full flowering and its end in rambler roses in the years 2000-2016. The 1st day of flowering scale begins at 25th May. Different small letters indicate significant differences between cultivars and capital letters between years, according to the timing of the start of flowering. The Duncan’s test (α = 0.05) was used.
